# Autoimmune pulmonary alveolar proteinosis presenting peripheral ground‐glass opacities

**DOI:** 10.1002/rcr2.385

**Published:** 2018-11-13

**Authors:** Keishi Sugino, Masahiro Ando, Kiyoshi Mori, Eiyasu Tsuboi

**Affiliations:** ^1^ Department of Respiratory Medicine Jizankai Medical Fundation Tsuboi Cancer Center Hospital Koriyama Japan; ^2^ Department of Respiratory Medicine Toho University Omori Medical Center Tokyo Japan

**Keywords:** Autoimmune pulmonary alveolar proteinosis, granulocyte/macrophage colony‐stimulating factor antibody, peripheral ground‐glass opacities

## Abstract

Autoimmune pulmonary alveolar proteinosis should be considered in the differential diagnosis of peripheral ground‐glass opacities.

## Clinical Image

**Figure 1:**
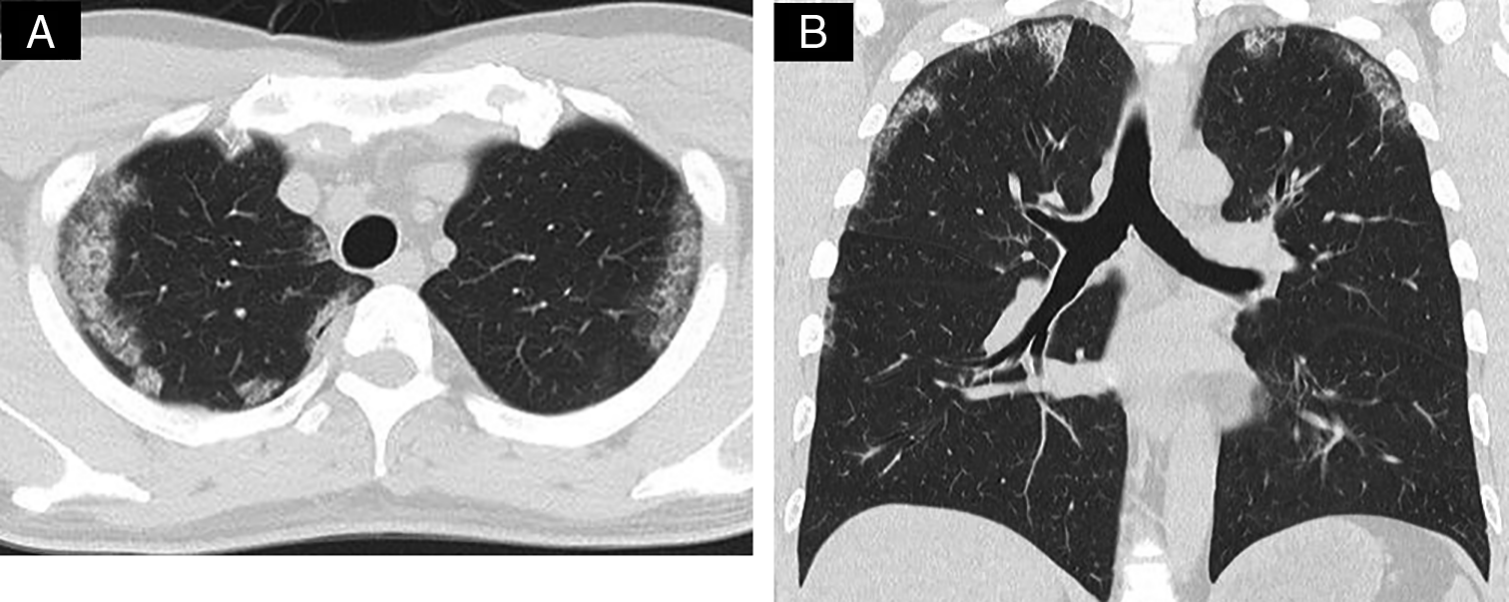
Chest computed tomography (CT) images demonstrates peripheral ground‐glass opacities in bilateral upper lobes. (A) Transverse section on chest HRCT, (B) coronal images of chest CT.

**Figure 2:**
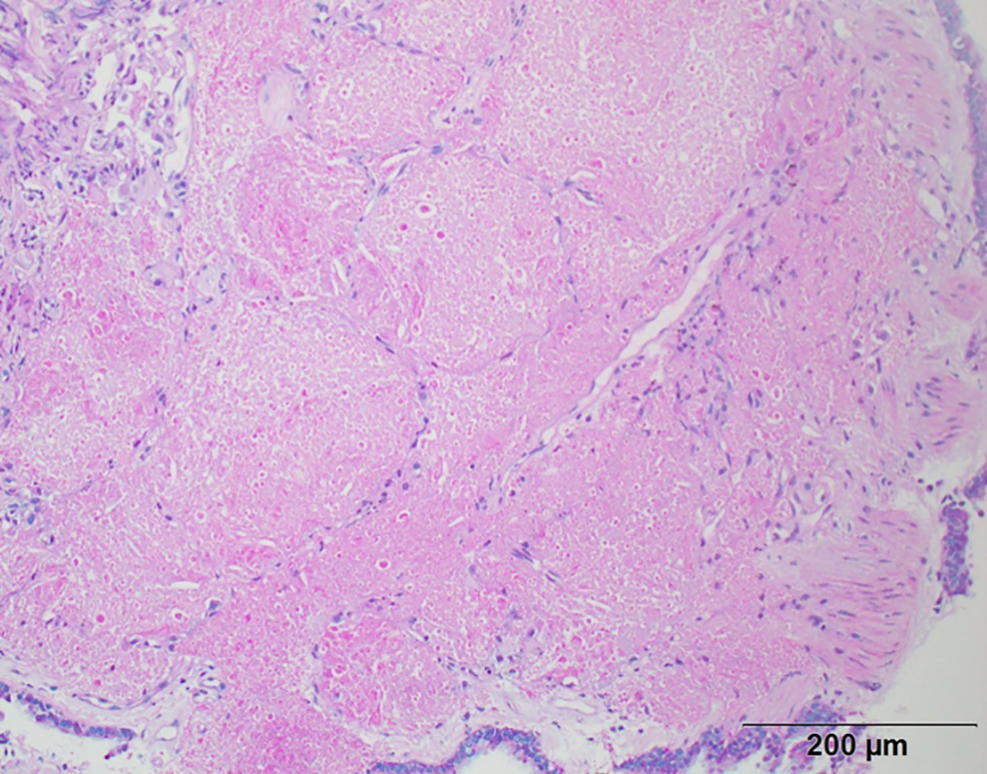
The transbronchial lung biopsy specimens shows the alveoli were filled with periodic acid‐Schiff (PAS)‐positive eosinophilic amorphous materials (PAS stain, scale bar = 200 μm).

A 41‐year‐old man with no history of smoking was referred to our hospital because of abnormalities on chest X‐ray as part of a routine health check‐up. He had had bronchial asthma at 23 years of age. Laboratory data on admission showed normal level of KL‐6 (300 U/mL) and elevation of SP‐D (273 ng/mL). The results of arterial blood gas analysis were pH, 7.41; PaCO_2_, 43.7 Torr; and PaO_2_, 79.4 Torr on room air. The pulmonary function test demonstrated normal respiratory functions with normal diffusing capacity. Chest computed tomography (CT) showed peripheral ground‐glass opacities (GGO) in the bilateral upper lobes (Fig. 1). Examination of bronchoalveolar lavage (BAL) fluid indicated alveolar macrophages, 47.2%; lymphocytes, 50.6%; neutrophils, 2.2%; and eosinophils, 0% with no turbidity. Total cells were increased, with a low CD4/CD8 ratio, 1.7. Cultures of sputum and BAL fluid were negative for fungal, bacterial, or mycobacterial pathogens. The transbronchial lung biopsy specimens showed that the alveoli were filled with periodic acid‐Schiff (PAS)‐positive eosinophilic amorphous materials (Fig. 2). The serum was positive for granulocyte/macrophage colony‐stimulating factor antibody (48.4 μg/mL). Consequently, the patient was diagnosed with autoimmune pulmonary alveolar proteinosis (aPAP), which developed in a never smoker presenting peripheral GGO. Satoh et al. 1 reported that pulmonary alveolar proteinosis (PAP) should be considered in the differential diagnosis of peripheral GGO. Patients with aPAP were often misdiagnosed as other interstitial lung diseases and treated with corticosteroids. As indicated by Akasaka et al. 2, corticosteroid therapy may worsen the disease severity in aPAP and increase the risk of infections.

### Disclosure Statement

Appropriate written informed consent was obtained for publication of this case report and accompanying images.
